# Whole blood gene expression and white matter Hyperintensities

**DOI:** 10.1186/s13024-017-0209-5

**Published:** 2017-09-18

**Authors:** Honghuang Lin, Claudia Satizabal, Zhijun Xie, Qiong Yang, Tianxiao Huan, Roby Joehanes, Chengping Wen, Peter J. Munson, Alexa Beiser, Daniel Levy, Sudha Seshadri

**Affiliations:** 1 National Heart Lung and Blood Institute’s and Boston University’s Framingham Heart Study, Framingham, MA USA; 2 Section of Computational Biomedicine, Department of Medicine, Boston University School of Medicine, 72 East Concord Street, B-616, Boston, MA 02118 USA; 3 Department of Neurology, Boston University School of Medicine, 72 East Concord Street, B-602, Boston, MA 02118 USA; 4 College of Basic Medical Science, Zhejiang Chinese Medical University, Hangzhou, Zhejiang Province China; 5 Department of Biostatistics, Boston University School of Public Health, Boston, MA USA; 6 Population Sciences Branch, Division of Intramural Research, National Heart, Lung, and Blood Institute, Bethesda, MD USA; 7 Mathematical and Statistical Computing Laboratory, Center for Information Technology, National Institute of Health, Bethesda, MD USA; 8 Hebrew Senior Life, 1200 Centre Street Room #609, Boston, MA 02131 USA

**Keywords:** White matter hyperintensities, Gene expression, Epidemiology

## Abstract

**Background:**

White matter hyperintensities (WMH) are an important biomarker of cumulative vascular brain injury and have been associated with cognitive decline and an increased risk of dementia, stroke, depression, and gait impairments. The pathogenesis of white matter lesions however, remains uncertain. The characterization of gene expression profiles associated with WMH might help uncover molecular mechanisms underlying WMH.

**Methods:**

We performed a transcriptome-wide association study of gene expression profiles with WMH in 3248 participants from the Framingham Heart Study using the Affymetrix Human Exon 1.0 ST Array.

**Results:**

We identified 13 genes that were significantly associated with WMH (FDR < 0.05) after adjusting for age, sex and blood cell components. Many of these genes are involved in inflammation-related pathways.

**Conclusion:**

Thirteen genes were significantly associated with WMH. Our study confirms the hypothesis that inflammation might be an important factor contributing to white matter lesions. Future work is needed to explore if these gene products might serve as potential therapeutic targets.

**Electronic supplementary material:**

The online version of this article (10.1186/s13024-017-0209-5) contains supplementary material, which is available to authorized users.

## Background

Magnetic resonance imaging (MRI) has been frequently used to identify abnormalities of white matter, detectable as white matter hyperintensities (WMH) [1, 2]. The prevalence of these white matter lesions increases with aging [3–5], and they typically happen long before the onset of clinically manifest neurological conditions [6]. WMH are observed in both apparently healthy individuals [2, 7, 8] and in individuals with stroke and dementia [9]. They have been associated with the risk of a variety of neurological diseases and other adverse outcomes, such as dementia [10–12], cognitive dysfunction [5, 9, 13–15], cardiovascular diseases [7], stroke [16–19], and mortality [20, 21]. The progression of WMH has also been used as a biomarker for predicting outcomes following stroke [22] and as a surrogate endpoint for clinical trials of cerebral small-vessel disease [23].

The pathophysiology of WMH remains poorly understood. Cardiovascular disease risk factors such as hypertension and diabetes are also risk factors for WMH, and small vessel arteriosclerotic disease is thought to be a key mechanism leading to WMH. In recent years, increasing evidence has suggested that chronic inflammation and glial proliferation might also be involved in the pathogenesis of WMH [24]. Moreover, it has been shown that WMH is highly heritable, with heritability estimates ranging from 55 to 73% [25–27]. Several genetic loci have been identified to be associated with WMH [28–31], however, they explain only a small portion of the observed heritability.

Gene expression has proved to be an important intermediate phenotype that helps to bridge genetic variations with many phenotypic traits [32–34]. Xu et al. [35] examined the association of gene expression in blood with WMH. Twenty participants with extensive WMH and 18 participants with minimum WMH were enrolled in the study. A total of 241 genes were found to be differentially expressed (*P* < 0.005 and at least 1.2 fold difference), many of which are involved in inflammation, oxidative stress, detoxification and hormonal responses. Another study by Simpson et al. [36] examined the association of gene expression in postmortem central nervous system tissue with WMH. Seven participants with WM lesions and 7 participants without lesions were enrolled in the study, and 502 genes were found to be differentially expressed, including genes involved in immunity and ion transport.

These studies have demonstrated that differential gene expression was associated with WMH. However, they examined small samples of highly selected participants, which limits their generalizability. The objective of this study is to assess the association of gene expression with WMH in over 3000 participants from the Framingham Heart Study (FHS).

## Methods

### Study samples

The FHS is a community-based, prospective study initiated in 1948 that aimed to investigate cardiovascular disease and its risk factors in the community. Three generations of participants have been enrolled, and multiple examinations have been performed with an interval of 2 to 8 years [37–39]. At each clinical examination, participants go through extensive physical examination, lifestyle and medical history interview. This analysis is restricted to the second generation (Offspring) of participants who attended the eighth Offspring examination (2005–2008), and the Third Generation participants who attended the second examination (2008–2011). All participants gave written informed consent, and the study was approved by the institutional review board at the Boston University Medical Center.

### WMH measurement

The details for the MRI protocol in FHS have been described previously [9, 40]. In brief, MRI was performed on a 1.5 Tesla Siemens Avanto scanner. Fluid attenuated inversion recovery (FLAIR) sequences were used for the measurement of WMH. The segmentation and quantification of WMH was performed using a semi-automated procedure [41], which has shown high inter-rater reliability [42]. Total intracranial volume (TCV), based on FLAIR, was quantified using the Quanta 2 software package [41].

### RNA extraction and gene expression profiling

Total RNA was isolated from frozen PAXgene blood tubes (PreAnalytiX, Hombrechtikon, Switzerland) and amplified using the WT-Ovation Pico RNA Amplification System (NuGEN, San Carlos, CA) according to the manufacturers’ standard operating procedures. The obtained cDNA was hybridized to the Affymetrix Human Exon 1.0 ST Array (Affymetrix, Inc., Santa Clara, CA). The raw data were quantile-normalized and log2 transformed, followed by summarization using Robust Multi-array Average [43]. The gene annotations were obtained from Affymetrix NetAffx Analysis Center (version 31). We excluded transcript clusters that were not mapped to RefSeq transcripts, resulting in 17,873 distinct transcripts (17,324 distinct genes) for downstream analysis.

Given that the gene expression was measured from whole blood, the proportion of different cell types might affect gene expression. However, only 2181 participants from the Third Generation cohort had measured cell counts, of which 1225 were included in the current study. For the remaining participants, we used the partial least square method to estimate the cell counts from those with measured cell counts based on the gene expression data [44]. The percentages of each imputed cell type were then normalized, where the negative predicted values were set to 0 and the sum of the percentages for all cell types was set as 100%. Cross-validated estimates of prediction accuracy (R2) were 0.61, 0.41, 0.25, 0.83, 0.83, 0.81, 0.89, and 0.25, for white blood cell counts, red blood cell counts, platelet counts, neutrophil percent, lymphocyte percent, monocyte percent, eosinophil percent, and basophil percent, respectively.

### Statistical analyses

WMH mesures were log transformed to reduce the skewness of its distribution. Linear mixed effects models were used to test the association between gene expression and WMH volumes, treating the expression of each gene as the dependent measure, and the log-transformed WMH measure as the exposure. The analyses were adjusted for age, sex, and TCV. We also adjusted for the differential cell counts using a fixed effect factor, and for familial relatedness by implementing a random variance-covariance matrix.

In our secondary analyses, we additionally adjusted for smoking, body mass index, systolic blood pressure, diastolic blood pressure, hypertension treatment, total cholesterol, HDL cholesterol, and triglyceride.

In order to correct for multiple testing, we used false discovery rate (FDR) [45], which is defined as the number of incorrectly rejected hypotheses divided by the total number of rejected hypotheses. Significant associations were defined as those with FDR < 0.05. All the analyses were performed using the “lmekin” R package (www.r-project.org/).

### Overlap with GWAS loci

The summary statistics of GWAS association was obtained from a meta-analysis of participants from multiple ancestries [46]. Four genetic loci were significantly associated with WMH. At each locus, we obtained all SNPs with *P*-value less than 5 × 10^−8^ (defined as GWAS SNPs). We then examined if any of GWAS SNPs was associated with gene expression in blood using FHS expression quantitative trait loci (eQTL) database [44]. An eGene was defined if its expression was associated with at least one of GWAS SNPs (FDR < 0.05). The association of each of the eGenes with WMH was then examined as described in the previous section.

We also queried the GWAS catalog [47] and extracted variants significantly associated with stroke or dementia (*P* < 5 × 10^−8^). For each of these variants, we searched its eGene, and examined the association of eGenes as described in the previous section.

### Construction of gene interaction subnetwork associated with WMH

A dense module searching strategy [48] was used to identify modules enriched with WMH-related genes. The experimentally validated interactions between genes were obtained from the PINA database [49]. Before the searching, each gene was assigned a score to represent its association with WMH. The module searching started with a seed gene that was significantly associated with WMH (FDR < 0.05). Neighboring genes were then added sequentially to the module if the addition increased the overall module score [50], which was defined as ${Z}_m=\frac{\sum {g}_i}{\sqrt{k}}$, where k is the number of genes in the module, and *g*_*i*_ is the score of the gene i. The searching stopped if no more genes could be added.

## Results

The current study includes 1397 eligible participants from the Offspring Cohort (mean age 66.4 ± 9.0 years, 54.2% women) and 1851 participants from the Third Generation Cohort (mean age 48.0 ± 8.5 years, 54.0% women) who had both gene expression and WMH measured. The descriptive characteristics of the participants are provided in Table 1.

**Table 1:** Baseline characteristics of the study participants

Characteristics	Offspring cohort (*n* = 1397)	Third generation cohort (*n* = 1851)
Women, *n* (%)	757 (54.2%)	1000 (54.0%)
Age, years (mean ± SD)	66.4 ± 9.0	48.0 ± 8.5
WMH, median (25th, 75th percentile)	2.41 (1.25, 4.69)	1.28 (0.92, 1.75)
Ln(WMH + 1), median (25th, 75th percentile)	1.23 (0.81, 1.74)	0.82 (0.65, 1.01)
Total cranial volume, median (25th, 75th percentile)	1222 (1143, 1307)	1254 (1170, 1344)
Current smoker, n (%)	97 (6.9%)	174 (9.4%)
Hypertension, n (%)	828 (59.3%)	135 (7.3%)
Body mass index (BMI), median (25th, 75th percentile)	27.52 (24.52, 30.78)	27.11 (23.98, 30.72)
Total cholesterol, median (25th, 75th percentile)	185 (162, 210)	184 (163, 207)
HDL, median (25th, 75th percentile)	56 (45, 68)	57 (47, 71)

### Association of Gene Expression with WMH volume

**Fig. 1:**
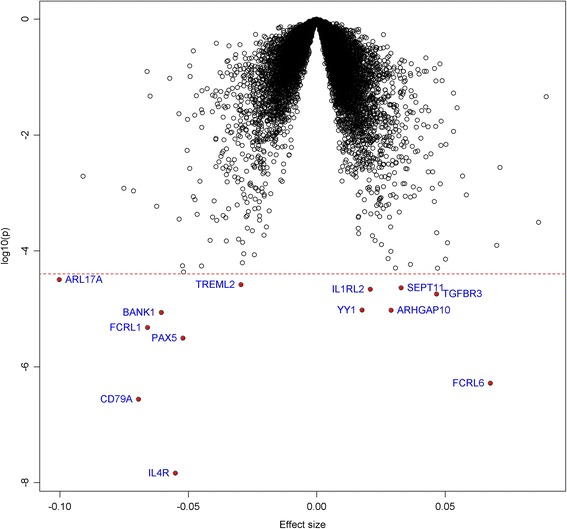
Volcano plot of gene expression associated with WMH. Each dot represents one gene. The x-axis represents the beta estimation (β) of each gene, whereas the y-axis represents the log_10_(*P*). Positive effects represent that genes were positively associated with WMH, whereas negative effects represent that the genes were negatively associated with WMH. The red dash line indicates FDR < 0.05. The 13 genes that reached significance cutoff were highlighted

As shown in Table 2 **,** a total of 13 genes were significantly associated with WMH (FDR < 5%). Six of them were upregulated, and the remaining seven genes were down-regulated. Figure 1 is the volcano plot showing the association between each gene with WMH. The most significant gene was *IL4R* (*P* = 1.5 × 10^−8^), which encodes the alpha chain of the interleukin 4 receptor. The result was similar after excluding articipants with stroke, dementia and vascular diseases (Additional file 1: Table S1).

**Table 2:** Most significant genes associated with WMH (FDR < 0.05)

Gene	Primary analysis^a^					Secondary analysis^a^			
	Effect size	SE^b^	P-value	FDR^c^	Direction of effect^d^	Effect size	SE^b^	*P*-value	Direction of effect^d^
*IL4R*	−0.055	0.010	1.5E-08	2.6E-04	↓	−0.050	0.010	3.6E-07	↓
*CD79A*	−0.069	0.013	2.7E-07	2.5E-03	↓	−0.067	0.014	1.0E-06	↓
*FCRL6*	0.068	0.013	5.2E-07	3.1E-03	↑	0.064	0.014	2.8E-06	↑
*PAX5*	−0.052	0.011	3.1E-06	1.4E-02	↓	−0.049	0.011	1.6E-05	↓
*FCRL1*	−0.066	0.014	4.8E-06	1.7E-02	↓	−0.059	0.015	5.8E-05	↓
*BANK1*	−0.060	0.014	8.6E-06	2.1E-02	↓	−0.057	0.014	3.4E-05	↓
*ARHGAP10*	0.029	0.007	9.4E-06	2.1E-02	↑	0.028	0.007	1.9E-05	↑
*YY1*	0.018	0.004	9.5E-06	2.1E-02	↑	0.019	0.004	3.5E-06	↑
*TGFBR3*	0.047	0.011	1.8E-05	3.6E-02	↑	0.044	0.011	6.4E-05	↑
*IL1RL2*	0.021	0.005	2.2E-05	3.7E-02	↑	0.020	0.005	6.1E-05	↑
*SEPT11*	0.033	0.008	2.3E-05	3.7E-02	↑	0.034	0.008	1.2E-05	↑
*TREML2*	−0.029	0.007	2.6E-05	3.9E-02	↓	−0.026	0.007	2.2E-04	↓
*ARL17A*	−0.100	0.024	3.2E-05	4.4E-02	↓	−0.097	0.025	7.8E-05	↓

^a^Primary analysis was adjusted for age, sex, total cranial volume and cohort, whereas secondary analysis was additional adjusted for smoking, body mass index, systolic blood pressure, diastolic blood pressure, hypertension treatment, total cholesterol, HDL cholesterol, and triglycerides

^b^SE: standard error

^c^FDR: false discovery rate

^d^Direction of effect: ↓ indicates decreased gene expression was associated with increased WMH, whereas ↑ indicates increased gene expression was associated with increased WMH

In the secondary analysis, we adjusted the model for additional clinical factors (see Methods). As shown in Table 2, most of top hits were still significant, although the association were slightly attenuated.

In order to assess potential effects of imputed cell counts, we performed a sensitivity analysis by including only individuals with measured cell counts, and compared the association of gene expression with WMH using either imputed or measured cell counts. As shown in Additional file 1: Figure S1, the statistics of associations were highly correlated using either the imputed and measured cell counts (R2=0.98), suggesting only marginal effect of imputed cell counts. We also additionally adjusted for the RNA integrity number in our statistical model, and found the results remained largely unchanged (Additional file 1: Table S2). We then performed another sensitive analysis by separating Offspring and Third Generation participants and assessed the association of gene expression with WMH separately. As shown in Additional file 1: Table S3**,** all 13 top genes had the same direction of association. In addition, the association in Offspring cohort showed relatively stronger effects than that in the Third Generation cohort, reflecting relatively older participants and more WMH volumes comparing to the Third Generation participants.

We also compared the top genes from the current study with previous studies. Among the 13 WMH-related genes, *SEPT11* was also reported to be associated with WMH in brain [36].

### Overlap with GWAS loci

In our recent GWAS that included more than 20,000 participants from diverse ancestries, we identified 4 genetic loci that were significantly associated with WMH [46]. As shown in Table 3, GWAS SNPs at these loci were associated with the expression of 17 genes (FDR < 0.05); three of them was nominally associated with WMH (*SEMA4A, UNC13D* and *WBP2*). As an example, the risk allele of rs2984613 was associated with decreased expression of *SEMA4A*, which was associated with increased WMH. We then searched all eQTLs for the 13 WMH-related genes, and found that these eQTLs were significantly enriched with variants associated with WMH [46] (*P* < 2.2e-16 by Kolmogorov–Smirnov test). Our results suggest that gene expression might serve as an intermediate phenotype that bridges genetic variations and WMH.

**Table 3:** Association of GWAS loci with gene expression. Three genes, *SEMA4A*, *UNC13D,* and *WBP2* were nominally associated with WMH

GWAS SNP	Risk allele^a^	Locus	*P* value (GWAS association with WMH) [46]	Association of SNPs with gene expression by eQTL analysis [44]			Association of eGene expression with WMH (current study)		
				eGene	*P* value	Direction on gene expression^b^	Effect size^a^	SE^c^	*P* value
rs4072479	C	17q25.1	4.6E-14	*WBP2*	1.0E-10	↓	**−0.020**	**0.010**	**4.6E-02**
rs8067275	T	17q25.1	1.9E-13	*MYO15B*	2.3E-05	↑	0.003	0.005	0.52
rs9894244	A	17q25.1	5.2E-10	*GALK1*	2.9E-06	↓	0.001	0.004	0.79
rs9894244	A	17q25.1	5.2E-10	*ITGB4*	2.9E-06	↓	−0.001	0.003	0.79
rs7216615	C	17q25.1	1.1E-09	*H3F3B*	9.2E-29	↓	−0.007	0.008	0.38
rs34143128	C	17q25.1	2.4E-09	*ACOX1*	9.1E-50	↓	0.003	0.009	0.75
rs1135889	A	17q25.1	8.8E-09	*UNC13D*	4.5E-11	↓	**−0.010**	**0.004**	**6.1E-03**
rs1135889	A	17q25.1	8.8E-09	*EXOC7*	9.1E-05	↓	−0.009	0.006	0.86
rs1135889	A	17q25.1	8.8E-09	*ZACN*	9.1E-05	↓	−0.001	0.005	0.86
rs2984613	C	1q22	2.0E-08	*C1orf85*	6.5E-23	↓	−0.003	0.007	0.65
rs2984613	C	1q22	2.0E-08	*TMEM79*	6.5E-23	↓	0.005	0.007	0.65
rs2984613	C	1q22	2.0E-08	*SMG5*	3.3E-20	↓	0.002	0.005	0.72
rs2984613	C	1q22	2.0E-08	*PAQR6*	1.6E-16	↓	0.001	0.006	0.89
rs2984613	C	1q22	2.0E-08	*PMF1*	7.3E-10	↑	−0.001	0.005	0.91
rs2984613	C	1q22	2.0E-08	*SEMA4A*	2.4E-08	↓	**−0.022**	**0.006**	**3.4E-04**
rs10883865	G	10q24.33	4.6E-08	*AS3MT*	7.9E-26	↓	0.011	0.010	0.30
rs10883865	G	10q24.33	4.6E-08	*USMG5*	7.7E-07	↑	0.015	0.011	0.19

^a^Risk allele respresnts the allele that was associated with increased risk of WMH

^b^Direction of effect: ↓indicates the risk allele was associated with decreased gene expression, whereas ↑ indicates the risk allele was associated with increased gene expression

^c^ *SE* standard error

Genes with *P*-value less than 0.05 were marked in bold text

We also examined if previously reported genetic loci for stroke and dementia were associated with 13 WMH-related genes in the current study. GWAS catalog was queried, and 142 genome-wide significant variants (*P* < 5 × 10^−8^) were found to be associated with dementia or stroke. We then searched these variants in FHS eQTL database [44] and found that they were associated with the expression of 70 eGenes; 7 of them were nominally associated with WMH, including *ARL17A, SYTL2, PTGDR, POLR2E, MS4A6A, GPR141,* and *RIN3*. Among them, *ARL17A* was the most significant one and it was associated with SNP rs2732703, which was recently found to be associated with Alzheimer’s disease among individuals without APOE ε4 allele [51].

### Pathway analysis

In order to examine the integrative effects of differentially expressed genes on the biological systems, we examined the enrichment of WMH-related genes in biological pathways using WebGestalt [52]. Given that only 13 genes reached the significance cutoff after correction for multiple testing, we expanded the selection and examined the enrichment of top 1% of genes associated with WMH (including 179 genes). Table 4 shows the top enriched biologic pathways (FDR < 0.05). Many of them are involved in the immune responses and apoptosis, such as antigen processing and presentation (FDR = 0.0019) and apoptosis (FDR = 0.0338).

**Table 4:** Most significant pathways enriched with top 1% genes associated with WMH. Two genes, ***IL4R*** and ***CD79A*** were highlighted as they both reached the significance cutoff WMH (FDR < 0.05)

Biological pathway	Total number of genes in the pathway	Ratio of enrichment	*P* value	FDR	Top 1% genes associated with WMH
Hematopoietic cell lineage	97	7.99	6.0E-06	0.0018	*HLA-DOB; HLA-DRA*; **IL4R**; *IL5RA; CD8A; CD19; CD22; CD37*
Antigen processing and presentation	77	8.81	1.3E-05	0.0019	*PSME3; HLA-DOB; HLA-DRA; KLRC3; KLRD1; CIITA; CD8A*
Inflammatory bowel disease (IBD)	65	8.95	5.0E-05	0.0050	*HLA-DOB; HLA-DRA*; **IL4R**; *RORA; STAT4; IL18R1*
Primary immunodeficiency	37	10.48	5.3E-04	0.0338	*CIITA; CD8A; CD19*; **CD79A**
Apoptosis	140	4.85	5.6E-04	0.0338	*BCL2L11; TUBA1B; CTSW; GZMB; BIRC3; TNFRSF10C; MAP3K14*
Graft-versus-host disease	41	9.46	7.9E-04	0.0399	*GZMB; HLA-DOB; HLA-DRA; KLRD1*
Cytokine-cytokine receptor interaction	265	2.93	0.005668	0.231	*CCR7*; **IL4R**; *IL5RA; PDGFRB; CCL4; CXCR5; TNFRSF10C; IL18R1*
B cell receptor signaling pathway	73	5.3	0.006633	0.231	*CD19; CD22; CD72*; **CD79A**
Allograft rejection	38	7.7	0.006858	0.231	*GZMB; HLA-DOB; HLA-DRA*
Type I diabetes mellitus	43	6.8	0.009672	0.285	*GZMB; HLA-DOB; HLA-DRA*

### Gene interaction network associated with WMH

**Fig. 2:**
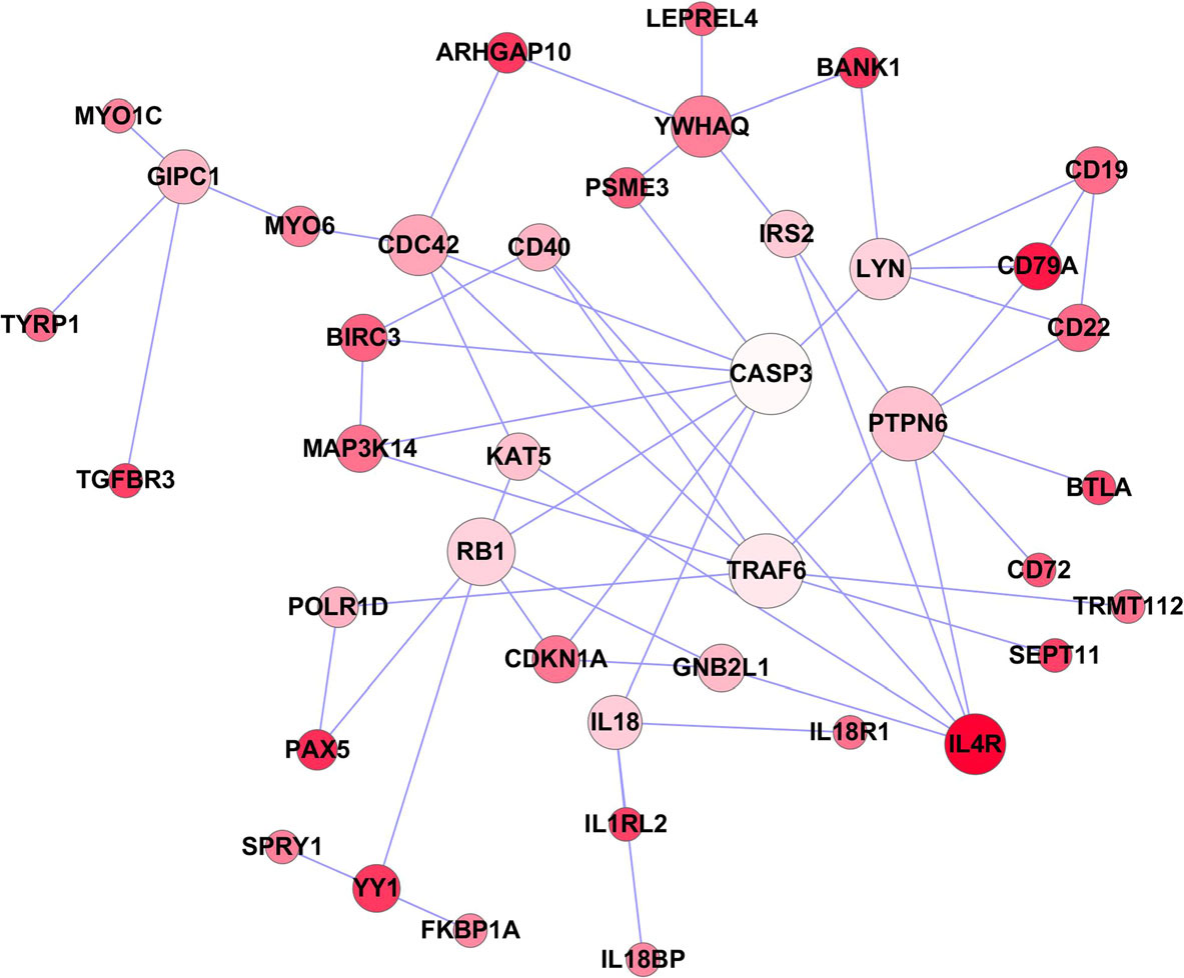
WMH-related subnetwork derived from protein-protein interaction. Each node represents one gene, wheras each edge represents the interaction between two genes. The nodes were colored to represent their association with WMH: red color represents strong association, and white color represents no association. The node size is proportional to the number of edges that the node connects to

We applied a dense module searching strategy [48] to construct a WMH-specific subnetwork and examined the interaction between top genes associated with WMH. Note that during the construction of subnetwork, genes with weak or no association with WMH might be also added to the subnetwork if the genes could interact with other significant genes, thus their inclusion would increase the overall score of the subnetwork (see Methods). As shown in Fig. 2, the subnetwork is consisted of 40 nodes and 57 edges, where each node represents one gene, and each edge represents the interaction between two genes. Many of these genes are involved in B cell receptor signaling pathway and Epstein-Barr virus infection. *CASP3* appears to be one of the pivotal genes in the network that was connected with 9 other genes, although itself was not associated with WMH (*P* = 0.79). Previously studies also have found that the activation of *CASP3* was observed in brain with ischemic lesions [53–55].

### Association with neuropsychological performance

The standardized neuropsychological performance testing was described previously [56–58]. Seven matrices were tested, including Hooper Visual Organization Test score, Logical Memories Delayed Recall score, Logical Memories Recognition score, Similarities Test score, Trails Test A score, Trails Test B score, and Trails B–A score. As shown in Additional file 1: Table S4, four genes were significantly associated with at least one cognitive matrix, including *BANK1* for logical memories recognition (*P* = 4.5 × 10^−4^), *TGFBR3* for Trails B–A score (*P* = 1.8 × 10^−3^) and Trails Test B score (*P* = 1.9 × 10^−3^), *FCRL1* for Hooper Visual Organization Test (*P* = 2.9 × 10^−3^) and *FCRL6* for Trails B–A score (*P* = 3.4 × 10^−3^). Our results suggest potential shared mechanisms between WMH and cognitive function.

## Discussion

Increasing evidence has suggested that WMH is an important surrogate of aging and cerebrovascular diseases [26, 28]. However, molecular mechanisms underlying WMH are still poorly understood. In this study, we performed transcriptome profiling on participants who lie across a wide age-range and were ascertained without specific clinical characteristics. We identified 13 genes that were significantly associated with WMH (FDR < 0.05) and examined their integrative effect by the network analysis. *CASP3* appears to be one of pivotal genes that interact with multiple genes associated with WMH.

Many of the WMH-related genes are involved in the immune response pathway, including the most significant gene, *IL4R* (*P* = 1.5 × 10^−8^) and others such as *CD79A*, *TGFBR3* and *IL1RL2*. A variety of studies have suggested a role of inflammatory processes in the development of cerebral large- and small-vessel disease [59, 60]. A high infectious burden is also associated with an increased risk of stroke [61]. Inflammatory markers, such as interleukin-6 and C-reactive protein, have been associated with the presence and progression of white matter lesions across multiple ancestries [24, 62]. Some of top genes are involved in tumorigenesis and Alzheimer type neurodegeneration, which is consistent with prior GWAS.

We found a single gene in the current study overlapping with those reported previously [35, 36]. The lack of overlapping might dues to several reasons. The gene expression in the current study was measured by the Affymetrix Exon 1.0 ST array instead of Affymetrix HU133 Plus 2.0 array, which interrogated different sets of genes with different probesets. In addition, our study focused on whole blood samples but with much larger sample size. Moreover, participants of current study are relatively young and generally healthy, which might represent the WMH burden in the general population. It should be noted that the expression in brain would be more relevant to WMH. However, it is impractical to examine the brain expression in a community-based cohort. We have developed a brain donation program [63], which will be a valuable resource to study brain gene expression profile in future.

We acknowledge several limitations of our study. Gene expression could vary from tissue to tissue over time, but we only measured it in whole blood during a single examination. So we could not study longitudinal changes in gene expression over time, and how this might be related to WMH. Less than half of the studied samples had measured cell counts, and the remaining samples used imputed cell counts, which could introduce some additional variations to our results. In addition, in this cross-sectional study of observational data, we could not infer causal relationships between gene expression and WMH; the observed gene expression changes could be a consequence of vascular injury, both systemic and in the brain. Moreover, all participants included in this study were exclusively adults of European descent. Thus it is unclear if our findings could be generalized to other ethnicities/age groups.

## Conclusions

In conclusion, we performed a large-scale profiling of gene expression in whole blood in a large community-based cohort, and identified 13 genes whose expression was associated with WMH. Our results are consistent with earlier reports that the immune response might be an important pathway to link gene expression and WMH. We also identified genes in glial proliferation and Alzheimer neurodegeneration pathways as potential links to WMH. Future studies with larger sample sizes and better techniques for measurement of gene expression such as RNA sequencing [64, 65] might uncover additional WMH-related genes and novel preventive and therapeutic targets for white matter lesions.

## Additional file

Additional file 1: Table S1. Association of top genes with WMH after excluding samples with stroke, dementia or vascular diseases. **Table S2.** Association of top genes with WMH after additionally adjusted for the RNA integrity number (RIN). **Table S3.** Separated analysis for participants from the Offspring cohort and the Third Generation cohort. **Table S4.** Association of top genes within cognitive performance. **Figure S1.** Correlation between the statistics of WMH associations derived from the imputed cell counts or the measured cell counts using those samples who have measured cell counts. X-axis represents the statistics derived from measured cell counts, while y-axis represents the statistics derived from the imputed cell counts. Strong correlation was observed (R2 = 0.984), suggesting only marginal effect of imputed cell counts. (PDF 187 kb)

## Abbreviations

eQTL: Expression quantitative trait loci
FDR: False discovery rate
FHS: Framingham Heart Study
GWAS: Genome-wide association study
TCV: Total intracranial volume
WMH: White matter hyperintensities
