# Supplementary material for: Whole blood gene expression and white matter Hyperintensities
Source: Mol Neurodegener. 2017 Sep 18;12:67. doi: 10.1186/s13024-017-0209-5 (PMC5604498; doi:10.1186/s13024-017-0209-5)

Whole Blood Gene Expression and White Matter Hyperintensities

Supplemental Table 1. Association of top genes with WMH after excluding samples with stroke, dementia or vascular diseases

Gene	Effect size	SE*	*P*-value
IL4R	-0.050	0.011	3.8E-06
CD79A	-0.068	0.015	6.2E-06
FCRL6	0.063	0.015	2.8E-05
PAX5	-0.051	0.012	4.8E-05
FCRL1	-0.060	0.016	1.9E-04
BANK1	-0.056	0.015	2.1E-04
ARHGAP10	0.027	0.007	2.0E-04
YY1	0.019	0.005	1.8E-05
TGFBR3	0.044	0.012	3.1E-04
IL1RL2	0.018	0.006	8.8E-04
SEPT11	0.034	0.009	6.2E-05
TREML2	-0.023	0.008	2.8E-03
ARL17A	-0.089	0.027	1.0E-03

*SE: standard error

Supplemental Table 2. Association of top genes with WMH after additionally adjusted for the RNA integrity number (RIN)

Gene	Effect size	SE*	*P*-value
IL4R	-0.054	0.010	2.0E-08
CD79A	-0.070	0.013	2.3E-07
FCRL6	0.067	0.013	5.7E-07
PAX5	-0.052	0.011	3.4E-06
FCRL1	-0.065	0.014	5.5E-06
BANK1	-0.061	0.014	7.6E-06
ARHGAP10	0.029	0.007	1.0E-05
YY1	0.017	0.004	1.2E-05
TGFBR3	0.047	0.011	1.4E-05
IL1RL2	0.021	0.005	2.7E-05
SEPT11	0.033	0.008	2.2E-05
TREML2	-0.030	0.007	2.1E-05
ARL17A	-0.098	0.024	4.1E-05

*SE: standard error

Supplemental Table 3. Separated analysis for participants from the Offspring cohort and the Third Generation cohort

Gene	Offspring cohort (n=1397)			Third generation cohort (n=1851)		
	Effect size	SE*	P-value	Effect size	SE*	P-value
IL4R	-0.049	0.012	6.8E-05	-0.055	0.020	6.4E-03
CD79A	-0.065	0.017	1.9E-04	-0.090	0.028	1.5E-03
FCRL6	0.063	0.018	3.0E-04	0.042	0.028	1.3E-01
PAX5	-0.051	0.015	4.6E-04	-0.057	0.023	1.3E-02
FCRL1	-0.056	0.019	2.6E-03	-0.079	0.031	9.6E-03
BANK1	-0.039	0.017	2.2E-02	-0.115	0.029	9.1E-05
ARHGAP10	0.030	0.008	4.3E-04	0.017	0.014	2.1E-01
YY1	0.022	0.005	9.0E-06	0.010	0.009	2.4E-01
TGFBR3	0.050	0.014	3.2E-04	0.002	0.023	9.3E-01
IL1RL2	0.019	0.006	8.6E-04	0.030	0.011	7.3E-03
SEPT11	0.031	0.010	1.2E-03	0.015	0.017	3.8E-01
TREML2	-0.020	0.008	1.3E-02	-0.046	0.016	3.3E-03
ARL17A	-0.114	0.031	2.5E-04	-0.027	0.054	6.1E-01

*SE: standard error

Supplemental Table 4. Association of top genes within cognitive performance

Trait+	Gene	Effect size	SE*	P-value
HVOT	*FCRL1*	0.0086	0.0029	**2.9E-03**
HVOT	*PAX5*	0.0064	0.0022	4.1E-03
HVOT	*CD79A*	0.0071	0.0027	8.9E-03
HVOT	*BANK1*	0.0069	0.0027	0.01
HVOT	*ARL17A*	0.0104	0.0049	0.03
HVOT	*IL4R*	0.0040	0.0019	0.04
HVOT	*TGFBR3*	-0.0034	0.0022	0.11
HVOT	*FCRL6*	-0.0037	0.0027	0.17
HVOT	*IL1RL2*	0.0005	0.0010	0.64
HVOT	*YY1*	-0.0004	0.0008	0.65
HVOT	*SEPT11*	-0.0006	0.0016	0.70
HVOT	*TREML2*	0.0003	0.0014	0.84
HVOT	*ARHGAP10*	-0.0003	0.0013	0.84
LMd	*PAX5*	0.0034	0.0015	0.02
LMd	*ARHGAP10*	-0.0018	0.0009	0.04
LMd	*IL4R*	0.0021	0.0013	0.12
LMd	*BANK1*	0.0026	0.0018	0.16
LMd	*FCRL6*	-0.0024	0.0018	0.18
LMd	*CD79A*	0.0023	0.0018	0.21
LMd	*FCRL1*	0.0022	0.0019	0.27
LMd	*ARL17A*	0.0034	0.0033	0.30
LMd	*YY1*	0.0005	0.0005	0.34
LMd	*SEPT11*	-0.0008	0.0010	0.44
LMd	*IL1RL2*	0.0005	0.0007	0.46
LMd	*TGFBR3*	-0.0010	0.0015	0.47
LMd	*TREML2*	-0.0001	0.0010	0.96
LMr	*BANK1*	0.0187	0.0053	**4.5E-04**
LMr	*PAX5*	0.0102	0.0044	0.02
LMr	*CD79A*	0.0095	0.0053	0.07
LMr	*ARHGAP10*	-0.0044	0.0025	0.08
LMr	*SEPT11*	-0.0052	0.0031	0.09
LMr	*ARL17A*	-0.0115	0.0096	0.23
LMr	*FCRL1*	0.0059	0.0057	0.30
LMr	*TREML2*	0.0028	0.0028	0.32
LMr	*IL1RL2*	0.0017	0.0019	0.39
LMr	*IL4R*	-0.0026	0.0038	0.50
LMr	*FCRL6*	-0.0028	0.0054	0.60
LMr	*TGFBR3*	-0.0015	0.0043	0.73
LMr	*YY1*	-0.0001	0.0016	0.95
SIM	*BANK1*	0.0057	0.0021	7.3E-03

Trait[+]	Gene	Effect size	SE*	P-value
SIM	*SEPT11*	-0.0029	0.0012	0.02
SIM	*ARL17A*	0.0081	0.0038	0.03
SIM	*PAX5*	0.0034	0.0018	0.05
SIM	*TGFBR3*	-0.0031	0.0017	0.07
SIM	*FCRL1*	0.0040	0.0022	0.08
SIM	*FCRL6*	-0.0037	0.0021	0.08
SIM	*TREML2*	0.0019	0.0011	0.09
SIM	*ARHGAP10*	-0.0017	0.0010	0.10
SIM	*IL4R*	0.0020	0.0015	0.18
SIM	*CD79A*	0.0027	0.0021	0.20
SIM	*YY1*	0.0006	0.0006	0.36
SIM	*IL1RL2*	0.0003	0.0008	0.68
TrA	*PAX5*	-0.0701	0.0259	6.8E-03
TrA	*YY1*	-0.0211	0.0094	0.02
TrA	*CD79A*	-0.0672	0.0309	0.03
TrA	*FCRL1*	-0.0649	0.0328	0.05
TrA	*TREML2*	-0.0293	0.0165	0.08
TrA	*IL4R*	-0.0366	0.0225	0.10
TrA	*BANK1*	-0.0443	0.0310	0.15
TrA	*ARHGAP10*	-0.0175	0.0148	0.24
TrA	*IL1RL2*	0.0125	0.0114	0.27
TrA	*FCRL6*	-0.0137	0.0314	0.66
TrA	*TGFBR3*	0.0102	0.0250	0.68
TrA	*ARL17A*	0.0156	0.0553	0.78
TrA	*SEPT11*	0.0016	0.0180	0.93
TrB	*TGFBR3*	0.0186	0.0060	**1.9E-03**
TrB	*FCRL6*	0.0201	0.0075	7.4E-03
TrB	*SEPT11*	0.0106	0.0043	0.01
TrB	*ARHGAP10*	0.0068	0.0035	0.06
TrB	*IL4R*	-0.0083	0.0054	0.12
TrB	*TREML2*	-0.0060	0.0039	0.13
TrB	*FCRL1*	-0.0114	0.0079	0.15
TrB	*BANK1*	-0.0082	0.0075	0.27
TrB	*PAX5*	-0.0058	0.0062	0.35
TrB	*ARL17A*	-0.0108	0.0136	0.42
TrB	*YY1*	0.0013	0.0022	0.57
TrB	*IL1RL2*	0.0014	0.0027	0.60
TrB	*CD79A*	-0.0026	0.0074	0.73
TrB_TrA	*TGFBR3*	0.0200	0.0064	**1.8E-03**
TrB_TrA	*FCRL6*	0.0235	0.0080	**3.4E-03**
TrB_TrA	*SEPT11*	0.0116	0.0046	0.01
TrB_TrA	*ARHGAP10*	0.0089	0.0038	0.02

Trait+	Gene	Effect size	SE*	P-value
TrB_TrA	*IL4R*	-0.0077	0.0058	0.18
TrB_TrA	*TREML2*	-0.0052	0.0042	0.22
TrB_TrA	*YY1*	0.0029	0.0024	0.23
TrB_TrA	*FCRL1*	-0.0095	0.0084	0.26
TrB_TrA	*ARL17A*	-0.0135	0.0145	0.35
TrB_TrA	*BANK1*	-0.0071	0.0080	0.37
TrB_TrA	*PAX5*	-0.0023	0.0066	0.73
TrB_TrA	*IL1RL2*	0.0006	0.0029	0.83
TrB_TrA	*CD79A*	0.0005	0.0079	0.95

+HVOT: Hooper Visual Organization Test score; LMd: Logical Memory Delayed Recall score; LMr: Logical Memories Recognition score; SIM: Similarities Test score; TrA: Trails Test A score; TrB: Trails Test B score; TrB_TrA: Trails B–A score

*SE: standard error

Supplemental Figure 1. Correlation between the statistics of WMH associations derived from the imputed cell counts or the measured cell counts using those samples who have measured cell counts. X-axis represents the statistics derived from measured cell counts, while y-axis represents the statistics derived from the imputed cell counts. Strong correlation was observed (R^2=0.984), suggesting only marginal effect of imputed cell counts.

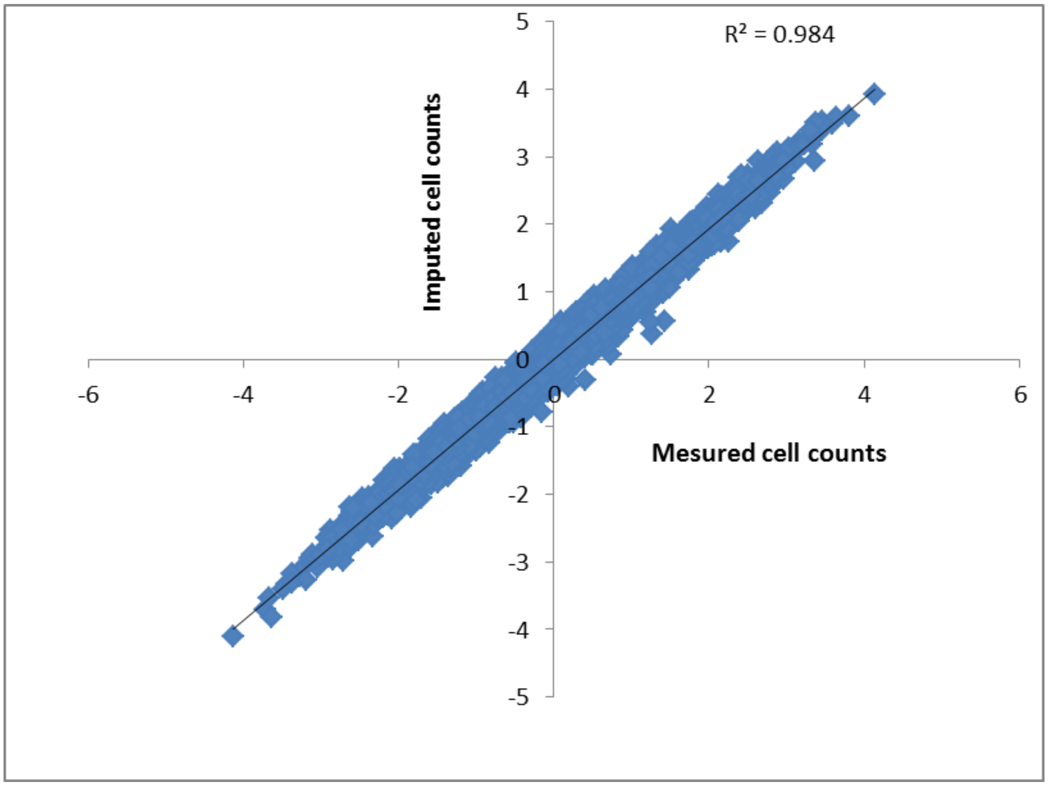

Supplement: Additional file 1: Table S1. — Association of top genes with WMH after excluding samples with stroke, dementia or vascular diseases. Table S2. Association of top genes with WMH after additionally adjusted for the RNA integrity number (RIN). Table S3. Separated analysis for participants from the Offspring cohort and the Third Generation cohort. Table S4. Association of top genes within cognitive performance. Figure S1. Correlation between the statistics of WMH associations derived from the imputed cell counts or the measured cell counts using those samples who have measured cell counts. X-axis represents the statistics derived from measured cell counts, while y-axis represents the statistics derived from the imputed cell counts. Strong correlation was observed (R2 = 0.984), suggesting only marginal effect of imputed cell counts. (PDF 187 kb) [file 13024_2017_209_MOESM1_ESM.pdf]
